# Clamp loader ATPases and the evolution of DNA replication machinery

**DOI:** 10.1186/1741-7007-10-34

**Published:** 2012-04-20

**Authors:** Brian A Kelch, Debora L Makino, Mike O'Donnell, John Kuriyan

**Affiliations:** 1 Department of Molecular and Cell Biology, University of California, Berkeley, CA 94720, USA; 2 Department of Chemistry, University of California, Berkeley, CA 94720, USA; 3 California Institute for Quantitative Biosciences, University of California, Berkeley, CA 94720, USA; 4 Howard Hughes Medical Institute, University of California, Berkeley, CA 94720, USA; 5 Physical Biosciences Division, Lawrence Berkeley National Laboratory, Berkeley, CA 94720, USA; 6 Howard Hughes Medical Institute, Rockefeller University, New York, NY 10021, USA; 7 Max-Planck Institute of Biochemistry, Department of Structural Cell Biology, D-82152 Martinsried, Germany

## Abstract

Clamp loaders are pentameric ATPases of the AAA+ family that operate to ensure processive DNA replication. They do so by loading onto DNA the ring-shaped sliding clamps that tether the polymerase to the DNA. Structural and biochemical analysis of clamp loaders has shown how, despite differences in composition across different branches of life, all clamp loaders undergo the same concerted conformational transformations, which generate a binding surface for the open clamp and an internal spiral chamber into which the DNA at the replication fork can slide, triggering ATP hydrolysis, release of the clamp loader, and closure of the clamp round the DNA. We review here the current understanding of the clamp loader mechanism and discuss the implications of the differences between clamp loaders from the different branches of life.

## 

High-speed replication of chromosomal DNA requires the DNA polymerase to be attached to a sliding clamp (known as proliferating cell nuclear antigen, or PCNA, in eukaryotes) that prevents the polymerase from falling off DNA [1,2]. In all cells and in some viruses, the clamp is a ring-shaped protein complex that encircles DNA, forming a sliding platform on which DNA polymerases and other proteins that move along DNA are assembled. Sliding clamps play a part in DNA replication, DNA repair, cell cycle control and modification of chromatin structure [3,4], and defects in several clamp-associated factors are associated with cancer and other disorders caused by abnormalities in DNA replication and repair [5].

Because sliding clamps encircle DNA but do not interact tightly with it, they can slide along the double helix by diffusion [6-9]. Sliding clamps from different branches of life have different subunit stoichiometry (they are dimers in bacteria [10] and trimers in eukarya, archaea and bacteriophage [11-15]) and their sequences have diverged beyond recognition. Nevertheless, their structures are remarkably similar. The conserved structure is an elegant symmetrical elaboration of a simple β-α-β motif, repeated 12 times around a circle [10,14]. The circular geometry is broken when the clamp is opened for loading onto DNA, but the elegance is retained during the loading step as the clamp assumes a helical symmetry that reflects the helical symmetry of DNA (see below).

The increase in the processivity and speed of DNA synthesis when DNA polymerases are engaged to sliding clamps is very considerable. For example, in the absence of the clamp, the polymerase subunit of the bacterial replicase synthesizes DNA at a rate of about 10 base pairs per second [16] and is hardly processive. In contrast, the same polymerase subunit synthesizes 500 to 1,000 nucleotides per second when bound to the sliding clamp [17-19]. To consider a startling analogy based on scaling linear dimensions, if the bacterial replicase were a car, it would travel only about 5 to 10 miles per hour without the clamp and faster than the speed of sound with the clamp. The bacterial replicase has a processivity of about 10 base pairs in the absence of the clamp [20], but has an average processivity of approximately 80 kilobases when bound to the sliding clamp in the replisome [21]. To invoke another analogy based on scaling dimensions, if the polymerase were a tightrope walker, without the aid of the clamp only about 20 feet of the tightrope would be traversed before the polymerase 'walker' fell off. The clamp allows the polymerase to hold on to the DNA 'rope' without letting go, and now it would 'walk' almost 30 miles before falling off.

The enhancement in speed and processivity that the clamp confers on the polymerase would not be possible without the clamp loader, the less glamorous but much more hardworking handmaiden of the sliding clamp, which diligently loads the clamps onto primed DNA throughout the process of DNA replication. Together, the clamp and the clamp loader lie at the heart of the replisome - the DNA replication machinery, which, with the polymerases (leading and lagging strand), includes the helicases that unwind the double-stranded DNA ahead of the polymerase at the replication fork, the primase that synthesizes the RNA primer required for the initiation of DNA synthesis, and the single-strand DNA-binding proteins that prevent the DNA from re-annealing in the wake of the helicases (Figure 1). The clamp loader opens sliding clamps and places them on the DNA at the site of the primer-template junction in the correct orientation for polymerase to bind, both at the initiation of DNA synthesis on the leading strand and continually at the start of each Okazaki fragment on the lagging strand. Thus, the clamp loader is critical for the tight coupling of leading and lagging strand synthesis. Indeed, in bacteria the clamp loader acts physically to hold the leading and lagging strand polymerases together [22-25] so that the two polymerases progress in tandem, with the lagging strand wrapped around the replisome in trombone fashion [26]. How leading and lagging strand polymerases are coupled in eukaryotic DNA synthesis is not known, and this is one of the major open questions about the operation of the eukaryotic replisome.

**Figure 1:**
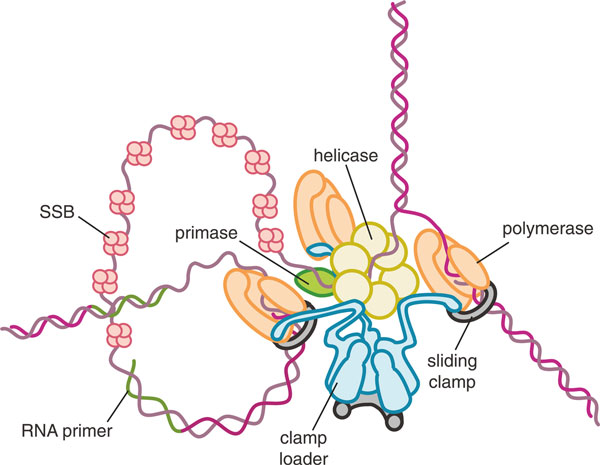
**Architecture of the bacterial replication fork**. The helicase is a homohexamer that encircles the lagging strand and binds directly to the primase synthesizing the primer RNA. The clamp loader acts to hold the replisome together by binding directly to the helicase as well as three polymerase subunits for simultaneous synthesis of the leading and lagging strands. The leading strand polymerase synthesizes DNA continuously, while the other two polymerases presumably cycle on and off the lagging strand, which is coated in single-strand binding protein (SSB). The polymerase subunits are attached to circular clamps that encircle duplex DNA for enhanced processivity and speed.

Despite the uncertainty in the precise architectural role of the clamp loader in the eukaryotic replisome, it is clear that sliding clamps are centrally important. The sliding clamp recruits the polymerase as well as other factors to the replication fork, including the chromatin-modifying proteins required to reassemble chromatin on the newly synthesized DNA [27,28].

## The clamp loader is a molecular switch operated by binding and hydrolysis of ATP

Clamp loaders are members of the AAA+ (ATPases associated with various cellular activities) family of ATPases [29] and derive from the same evolutionary root as helicases and other motors that work on DNA, many of which are also AAA+ ATPases. The role of AAA+ proteins is not restricted to DNA-dependent processes, and there is hardly an aspect of cellular function that does not have an AAA+ machine playing an important role. In architecture and mechanism, the AAA+ ATPases are related distantly to the F_1_-ATPase [30], and as with that energy-transducing machine, evolution has built AAA+ systems into the master plan of life. A comprehensive review of AAA+ ATPases is provided by Berger and Erzberger [31], to which the reader is referred for a thorough discussion of these ideas.

**Figure 2:**
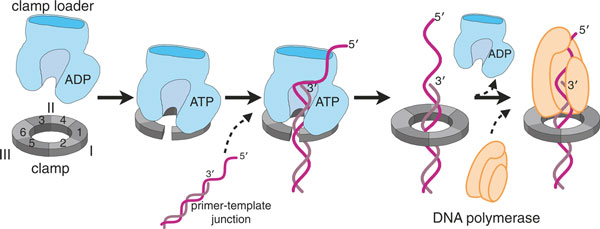
**Clamp loaders place sliding clamps at primer-template junctions for processive DNA replication**. When bound to ATP, clamp loaders are competent to bind and open the sliding clamp protein. This ternary complex can now bind to a primer-template junction, which activates the ATPase activity of the clamp loader. ATP hydrolysis causes the clamp loader to dissociate from the clamp and DNA, resulting in a loaded clamp that is competent for acting as a processivity factor for DNA polymerase. Figure adapted from [60].

Initially thought to be a motor [32-34], the clamp loader is now better thought of as a timing device or molecular switch [35], related conceptually and in molecular mechanism to small GTPases such as Ras [36]. The clamp loader must be bound to ATP in order to bind and open the clamp [37,38] and to bind primer-template DNA [39-41] (Figure 2). ATP hydrolysis is, however, not necessary for clamp opening, which is thought to depend simply on the affinity of the ATP-bound clamp loader for the open conformation of the clamp: in the ADP or empty state, the clamp loader has low affinity for the clamp [42,43]. The ATPase activity of the clamp loader is stimulated by binding both to the clamp and to DNA [39,40], and upon ATP hydrolysis the affinity of the clamp loader for both clamp and DNA is greatly diminished, leading to ejection of the clamp from the clamp loader.

This complex but fundamental mechanism is embodied in an assembly of surprisingly diverse composition from bacteria to eukaryotes.

## The structure of the clamp loader is more conserved than its composition

**Figure 3:**
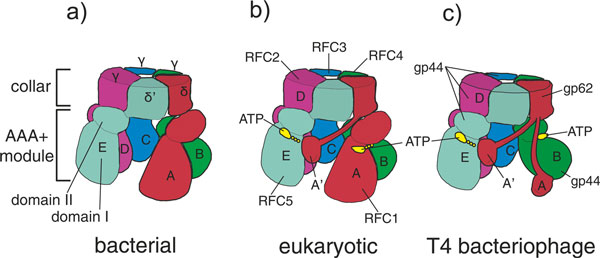
**Composition of clamp loaders from the different branches of life**. **(a) **Bacterial clamp loaders consist of three different proteins: δ, δ', and the τ or γ protein (γ, a truncation of the τ protein, is shown here). The δ protein is at the A position, with three copies of the ATPase subunits τ or γ at the B, C and D positions. The δ' protein sits at the E position. **(b) **Eukaryotic clamp loaders consist of five distinct proteins, RFC1 through RFC5. RFC1 lies at the A position, RFC4 at B, RFC3 at C, RFC2 at D and RFC5 at the E position. Eukaryotic A subunits contain an additional domain that bridges the gap between the A and E positions. **(c) **Bacteriophage clamp loaders consist of two distinct proteins. The gp62 protein, which lacks a AAA+ module but has an A' domain similar to that of eukaryotic clamp loaders, lies at the A position. The ATPase gp44 protein lies at the B, C, D, and E positions. Archaeal clamp loaders have a similar composition (see text for details). Figure adapted from [60].

Unlike other AAA+ ATPases, which are typically hexameric, all clamp loaders are composed of five subunits in a circular arrangement, with a gap between the first and the fifth subunit, at the position of the missing sixth subunit. The individual subunits are designated A through E, starting with the subunit at the open interface and proceeding counter-clockwise around the clamp loader in the standard view (Figure 3). Each subunit has three domains. The amino-terminal domain and its adjacent domain assume the fold of AAA+ proteins (with one exception to be explained later); the first structure to be determined for an AAA+ fold was one of the five *Escherichia coli *subunits in isolation [44]. The subunits are held in a ring by their carboxy-terminal domains, which together form a tight pentameric collar.

The clamp loader shares an essential aspect of its mechanism with other AAA+ complexes and other oligomeric ATPases, such as the F_1_-ATPase [30]: the binding of ATP brings together the inter-subunit interfaces, most notably the arginine finger residues that are essential for hydrolysis of ATP [45,46] (so called by analogy with the catalytic residue from the activators of small GTPases [47]). This conformational rearrangement also results in a spiral organization of the five amino-terminal regions of the clamp loader. The coupling of the suicidal binding of ATP to large-scale conformational change drives alterations in molecular organization that are necessary to hold the sliding clamp open and position it on the DNA.

Because clamp loaders are so fundamental to the replication process, it is no surprise that their structure and mechanism turn out to be highly conserved in all branches of life. This conservation has been somewhat difficult to appreciate, because the extensive biochemical analyses of bacterial, eukaryotic and bacteriophage clamps and clamp loaders have to a great extent proceeded independently in the past, and scientists working on these systems have used different conventions to identify the subunits. Adding to the confusion is the fact that, even though all clamp loaders have five essential subunits, the protein stoichiometry is different in clamp loaders from different branches of life. This confusion can be alleviated by using the simple A through E scheme for identifying the clamp loader subunits. Figure 3 is a schematic illustration of the bacterial, eukaryotic and bacteriophage clamp loaders showing the relationship of the different proteins they are composed of to the conserved subunit organization; and to help avoid confusion as we discuss the clamp loader mechanism on the basis of studies in different organisms, we describe below the three major variants.

**Figure 4:**
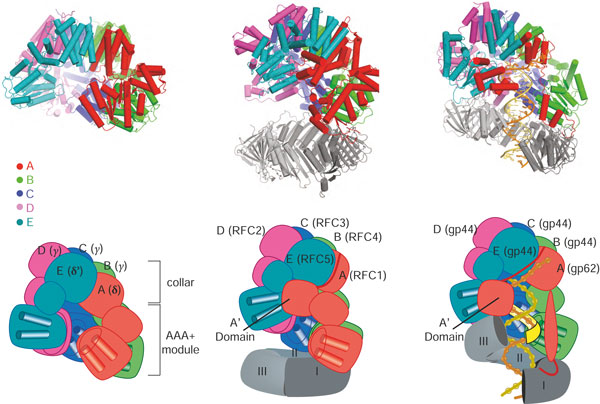
**Structures of clamp loaders from different branches of life and in different bound states**. The five subunits of the clamp loader - A, B, C, D and E - are shown in different colors. **(a) **The structure of the clamp loader of *E. coli *(known as the γ-complex) in the apo form (PDB code 1JR3) [114]. This structure illustrates the three conserved domains of clamp loader subunits. The two amino-terminal domains constitute the AAA+ module. The carboxy-terminal domains form a disc-like structure that holds the complex together as a tight pentamer. **(b) **Structure of the budding yeast clamp loader, replication factor-C (RFC), bound to the sliding clamp, PCNA, and an ATP analog (PDB code 1SXJ) [59]. ATP induces a spiral arrangement of the AAA+ modules. The clamp is not open in this structure, probably because of mutations in key interfacial residues (the arginine fingers) that disrupt the tight interactions between adjacent AAA+ modules and prevent hydrolysis of the ATP analog. **(c) **Structure of the T4 bacteriophage clamp loader bound to primer-template DNA, an open sliding clamp and ATP analog (PDB code 3U60) [60]. The duplex region of the primer-template junction is bound within the central chambers of the clamp loader and the sliding clamp, with the 5' single-stranded template extruded through the gap in the clamp loader where the missing sixth subunit would be. The AAA+ modules of the clamp loader, bridged by the ATP analog, form a spiral that perfectly matches the helical symmetry of DNA. Movies of the 3 structures that form the upper panel are available by clicking on them in the PDF version of this article. They are also available as additional files 1,2 and 3. (Adobe Reader Version 8 or higher required).

The bacterial clamp loader is formed from three essential subunits: δ, δ', and the τ or γ protein (Figures 3a and 4a). The δ and δ' proteins, which have no ATPase activity, are present in one copy each in the clamp loader, at the A and E positions, respectively. The B, C and D positions in the assembly are composed of either τ or (in *E. coli*) γ ATPase proteins. The γ protein is a truncated version of the τ protein that lacks the elements necessary for binding to the helicase or the polymerase. There is evidence that most replisomes *in vivo *contain the τ protein at the B, C and D positions, so that the replisome has three polymerase subunits bound [48,49] (Figure 1). Bacterial clamp loaders often have two accessory subunits (χ and ψ) that are not members of the AAA+ family [50] and are not necessary for the clamp loading process but couple the clamp loader to single-strand DNA-binding protein [51-53]. Binding of the ψ protein also induces a conformational change in the clamp loader that increases its affinity for DNA [54,55].

The eukaryotic clamp loader, Replication Factor C or RFC [56-58], is composed of five unique proteins, RFC1 through RFC5 (Figures 3b and 4b). The largest subunit, RFC1, is at the A position and contains an active ATPase site as well as an extra domain (called the A' domain) carboxy-terminal to the collar and that interacts with the AAA+ module of the E subunit [59], thus bridging the gap left by the missing sixth subunit. The other four positions in the clamp loader are occupied by similarly sized proteins: RFC4 at B, RFC3 at C, RFC2 at D, and the ATPase-incompetent RFC5 protein at the E position.

The bacteriophage and archaeal clamp loaders are both composed of two proteins each with one unique protein occupying the A position (gp62 in T4 bacteriophage and RFC-l in archaea) and identical ATPase subunits at the B, C, D and E positions (the gp44 protein in T4 bacteriophage and RFC-s in archaea) [11,60-62] (Figures 3b and 4c). While the archaeal clamp loader contains an active ATPase at the A position [63], the A subunit in the T4 bacteriophage clamp loader does not have a AAA+ fold [60]. Like the eukaryotic clamp loader, the A subunit of the T4 and archaeal clamp loaders contains an A' domain [60,62].

The bacteriophage clamp loader, whose structure seems to reflect a rather curious evolutionary history (we return to this briefly later), has played a particularly important part in the elucidation of the clamp loader mechanism. A recently determined structure of the T4 bacteriophage clamp loader bound to an ATP analog and in complex with the sliding clamp and primer template DNA [60] revealed a state of the system that we have long sought to visualize: an open clamp encircling DNA while in complex with an ATP-bound clamp loader. Another structure shows what happens when the loader hydrolyzes a single ATP molecule. Through a combination of the most recent T4 structures with previous structural and biochemical data, many general features of clamp loader structure and function can be placed in the context of detailed structural models for changes in conformation and the assembly of complexes.

## Recognition of the clamp during loading onto DNA

In three crystal structures of ATP-bound clamp loaders, the AAA+ modules can be seen to form a right-handed spiral [55,59,60]. The clamp binds 'under' the clamp loader in the 'standard view' (Figure 4b,c). Three-dimensional image reconstructions from electron microscopy of a clamp-bound archaeal clamp loader show that the loader holds the clamp in an open spiral form [62]. The crystal structure of the T4 clamp loader bound to the clamp and DNA confirms the generality of this interaction [60] (Figure 4c). Indeed, there is some evidence from molecular dynamics simulations that clamp proteins are inherently biased toward a right-handed spiral shape when opened [64,65], although not all simulations show this right-handed bias [66].

**Figure 5:**
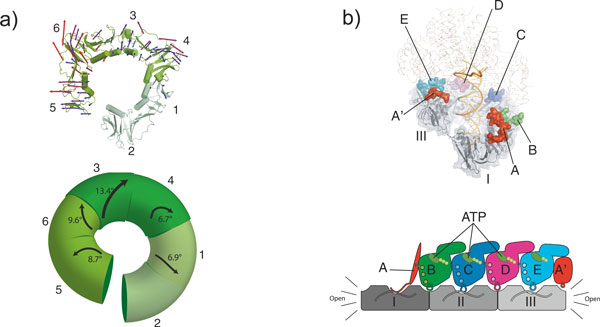
**Clamp loader interactions to open the sliding clamp**. **(a) **The clamp is held in a distorted conformation. In the top panel, displacement vectors compared from the closed planar structure (PDB code 1CZD) [13] to that of the open form (PDB code 3U60) [60] are mapped out onto the structure and are scaled up by a factor of 4 and color coded blue to red. In the bottom panel, the relative domain rotations from the closed planar structure to the open conformation are mapped onto the schematic, with the vector length and width scaled by the magnitude of rotation. Figures adapted from [60]. **(b) **Clamp loader contacts to the open sliding clamp. All five clamp loader subunits contact the open clamp. In the top panel, the clamp-interacting motifs of the clamp loader are shown with the rest of the structure displayed as a wireframe for clarity. In the bottom schematic diagram, the clamp loader AAA+ modules and the sliding clamp are shown from the side and flattened out onto the page so that all subunits can be viewed simultaneously. The contacts between the ATPs and arginine fingers direct the clamp loader to form a spiral assembly in which the AAA+ modules are arranged to perfectly match the symmetrically positioned binding clefts on the sliding clamp. Figure adapted from [60].

The right-handed spiral of the open clamp roughly matches the helical symmetry of DNA, with the clamp tracking along the minor groove of the DNA duplex. The right-handed spiral of the clamp can be described as a series of rigid-body rotations of the six domains present in the clamp (Figure 5a). The distortions of the clamp from its planar conformation are not uniform around the spiral. The largest distortion occurs between domains 3 and 4 of the clamp (a 13.4° rotation), which is the domain interface directly opposite the open interface. While it may seem counterintuitive that the domains nearest the opening show the least perturbation, a large rotation at the site opposite the broken interface is amplified around the ring, thus providing the greatest leverage for clamp opening.

The recent structures of the T4 clamp loader bound to an open clamp and primer template DNA indicate how loaders open a clamp [60]. The T4 clamp loader holds the clamp at six contact points: one each from the B, C, D, and E subunits, and two from the A subunit (the A domain and A' domain contact the clamp on either side of the open interface; Figures 4c and 5b.) Three of the contacts (from A, C and E subunits) occupy a hydrophobic pocket between the two domains of each subunit; this is the canonical site whereby clamps interact with other proteins [12,32,67-69]. The other clamp loader contacts (from B, D, and A') occupy grooves that lie at the interfaces between clamp subunits. In this way the clamp loader completely occludes the face on which the clamp binds other components of the replisome. This occlusion explains the observation that binding of DNA polymerase and clamp loader are mutually exclusive [70-72].

The B, C, D and E subunits of the T4 clamp loader are identical (see above), yet they can occupy two very different binding surfaces on the clamp: the canonical binding cleft or the inter-subunit crevice (Figure 5b). These subunits bind to the clamp through relatively non-specific van der Waals contacts, with limited hydrogen bonds and ionic interactions. The B, C, and D subunits of the bacterial clamp loader (also identical proteins, γ or τ) can also bind to two kinds of sites on the clamp. This raises the question of how the T4 and bacterial clamp loaders achieve proper alignment with the clamp, so that the ATP-driven conformational change in the clamp loader can be coupled appropriately to opening of the clamp. (Note that this question does not arise for eukaryotic clamp loaders, whose five subunits are distinct and can be specific for their requisite binding site.)

In both the T4 and the *E. coli *clamp loaders, this problem is solved by specific interactions between the clamp and the unique A subunit (Figure 5b). (The eukaryotic A subunit also makes specific contacts with the open end of the clamp.) In the case of the *E. coli *clamp loader, the A subunit interacts more tightly with the clamp than do any of the other subunits [73]; it binds through a helix and loop that insert snugly into a hydrophobic pocket, accompanied by specific hydrogen bonds and ions pairs between the A subunit and clamp [32]. Likewise, the A subunit of the T4 clamp loader has the largest interaction surface area of all its subunits and makes interactions that are specific for the deep hydrophobic binding cleft [60], consistent with biochemical studies that have shown that the A subunit is necessary for productive binding of the clamp [74,75].

The clamp loader, as we have seen, interacts with the clamp through the cooperative action of multiple interactions that appear to be weak. Most other clamp-binding proteins rely primarily on one tight interaction with the clamp. For example, most DNA polymerases [12,67,76,77] and the cell cycle regulator p21 [68] interact with the clamp primarily through a single, high-affinity binding site. The weak cooperative interactions between clamp loader and clamp are easily regulated to facilitate clamp loader ejection after loading the clamp onto DNA (see below). Proteins that interact primarily through one tight binding site allow the clamp to bind multiple partners at once, so that, as suggested in earlier studies, the clamp can be a sliding tool belt on DNA [78-80].

While the recently determined structures show how the clamp is held in the open state by the clamp loader, they do not address whether the clamp loader actively opens the clamp or simply traps a transiently open state. The T4 clamp appears to be dynamic and can open and close even in the absence of the loader [81-84], possibly explaining the short lifetime of the T4 clamp on DNA compared with cellular clamps [85]. Hence, the T4 clamp loader may not need to force the clamp open, but may simply trap it in the open state and close it around a primer-template junction in the correct orientation for polymerase action. Conversely, the *E. coli *clamp forms an extremely tight, closed structure in isolation [10,37,86] and has a long lifetime on DNA (t_1/2 _~ 1 h) [85]. Thus, bacterial clamp loaders are thought to open the clamp actively [37,86,87], probably through contacts from the A subunit that cause a conformational change in the clamp that destabilizes the interface between clamp subunits [32]. Therefore, clamp loaders from different organisms can be expected to behave differently: some clamps may need to be actively unloaded from DNA while others will fall off spontaneously. The same is true for clamp loaders with different functions - for example, the RFC-Ctf18 variant clamp loader, which is involved in sister chromatid cohesion [88,89], has been suggested to be a dedicated clamp unloader [90].

## The clamp loader and the clamp collaborate in specific binding of the clamp loader to primer-template junctions

**Figure 6:**
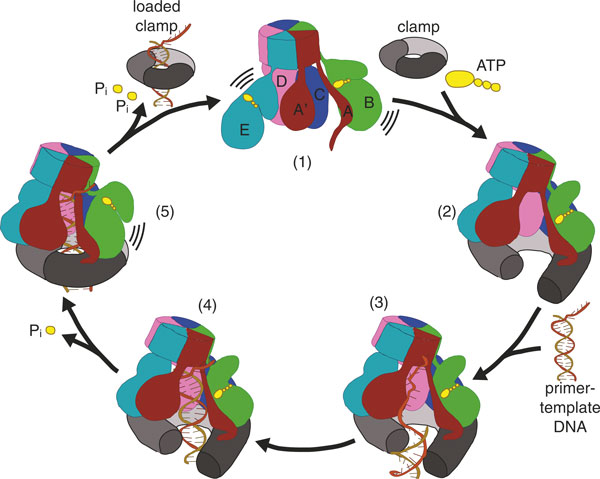
**A detailed model for clamp loading mechanism**. (1) Prior to ATP binding, the clamp loader AAA+ modules are not organized in a manner competent to bind the clamp. (2) Upon fully binding ATP, the clamp loader AAA+ modules assume a spiral shape that can hold the clamp in an open lockwasher conformation. (3) This binary complex is competent to bind to primer template DNA. We propose that the dimensions of the open clamp restrict DNA access to the central chamber such that only single-stranded regions or a major groove at a single-stranded/double-stranded junction can slip through the crack in the clamp. (4) The duplex region of primer-template DNA then slides up into the clamp loader central chamber, which activates the ATPase. (5) ATP hydrolysis or P_i _release initiates at the end of the AAA+ spiral, which allows the clamp to close. Further hydrolysis events cause release of the clamp loader from the clamp and DNA, resulting in a loaded clamp. Figure adapted from [60].

The clamp loader must position the sliding clamp on the DNA specifically at the primer-template junction where the polymerase is to be recruited. How the clamp loader recognizes the primer-template junction was first suggested by the structure of yeast clamp loader RFC bound to PCNA in the absence of DNA [59] (Figure 4b). The duplex segment of primer-template DNA is positioned in the central chamber of the clamp loader where it is stabilized by basic residues and amino-terminal helix dipoles lining the interior surface of the clamp loader spiral. Because the circular collar domains form a disc with no central cavity, it blocks the 3' end of the primer strand and in this way selects for DNA structures that can bend sharply and thus leave the interior of the clamp loader through the gap between the A and E subunits (in bacterial clamp loaders) or the A and A' domains (in the other clamp loaders) (Figures 3 and 6). The single-stranded template DNA at the primed template junction is such a flexible structure. This escape route for the template DNA is possible only because the clamp loader is pentameric, and not hexameric, as is typical for AAA+ machines: it is the gap where the sixth subunit would be that allows egress of the single-stranded template. The model in Figure 6 also shows how the binding of primed DNA by the clamp loader would automatically position the duplex region of a primer-template junction through the ring of the sliding clamp.

The clamp loader AAA+ spiral does not conform to B-form DNA, but matches instead the helical symmetry of duplex A-form DNA [55,60]. It thus forces the duplex DNA in the chamber into the A conformation. It is perfectly adapted to bind the RNA-DNA hybrid segment where an RNA primer is present, since RNA-DNA hybrids assume a conformation that closely resembles A-form DNA [91]. The principal interactions between the clamp loader and double helix are restricted to the template strand [55,60]. These features enable the clamp loader to bind either RNA-DNA or DNA-DNA primer-template junctions, a versatility that is important in the participation of clamp loaders in DNA repair, where the primer is DNA.

For DNA to bind in the central chamber of the clamp loader, it must enter through the clamp opening as well as the gap in the clamp loader spiral, which in the T4 clamp loader is flanked by the A' and A domains of the A subunit. The structures suggest, however, that both gaps are too narrow for either duplex DNA or a DNA-RNA hybrid to pass through. The clamp is only open by approximately 9 Å, and the gap between the A and A' domains of the clamp loader is as small as 14 Å [60]. (Eukaryotic and archaeal clamp loaders, with larger A domains, would be expected to have an even smaller gap between the A and A' domains.) Although the T4 structure already has DNA present, Förster resonance energy transfer experiments of the yeast clamp bound to the clamp loader have shown that the opening of the clamp is not significantly changed upon DNA binding [92].

To explain how the DNA-RNA hybrid at the primer-template junction gains access to the chamber of the clamp loader, we propose that single-stranded DNA, or possibly the major groove of duplex DNA directly adjacent to a primer-template junction, initially enters through the opening in the clamp (Figure 6). Once through the opening, the duplex region could then screw up into the interior of the clamp loader to occupy the central chamber, where it would stimulate ATPase activity and induce clamp loader ejection (see below). This feature may have important functional implications for the clamp loader mechanism at a replication fork, as the small opening may act as a filter for regions of DNA with single-stranded character, thereby aiding in the search for primer-template junctions.

## DNA-triggered ATP hydrolysis drives ejection of the clamp loader

In all clamp loaders tested thus far, ATPase activity is greatly enhanced by the binding of primer-template DNA [40,93-95]. Hydrolysis of ATP leads to closure of the clamp and ejection of the clamp loader from the clamp and DNA, leaving the clamp loaded onto DNA [37,40]. The DNA-dependent hydrolysis of ATP is a key feature of clamp loaders because it prevents futile cycles caused by premature release of a clamp before a primer-template junction is found.

The structural data suggest how DNA binding could play a role in switching on the ATPase activity of the clamp loader subunits. Earlier structural analyses of the bacterial and eukaryotic clamp loaders showed how the formation of tight intersubunit interfaces on ATP binding organizes the arginine finger at the catalytic center [55,59]. The availability of the T4 structure, along with other structures, suggests a possible allosteric switch mechanism, in which a DNA-binding residue (the switch residue) undergoes a DNA-dependent conformational change that appears to control the positioning of the catalytic glutamate in the Walker B motif [60]. This mechanism was suggested on the basis of sequence covariation to function in the eukaryotic clamp loaders [96], and is conceptually similar to the glutamate switch mechanism that has been proposed for other AAA+ proteins, in which a conserved asparagine holds the catalytic glutamate in an inactive conformation until ligand binds [97]. We now propose that this mechanism plays a role in ATPase activation in all clamp loaders. In the absence of DNA, the conserved basic switch residue (Arg383 in the yeast A subunit, Lys100 in *E. coli *B, C and D subunits, and Lys80 in the T4 B, C, D and E subunits) is tucked into the interior of the AAA+ module, where it interacts with the backbone of the Walker B residues and holds the catalytic glutamate in an inactive conformation [59]. Upon binding DNA, the switch residue interacts directly with the phosphate backbone of the DNA template strand and is released from the interior of the AAA+ module, allowing the catalytic glutamate to enter a conformation consistent with activation of water for hydrolysis of the γ-phosphate of ATP [55,60]. Mutational analysis of the switch residue and neighboring residues supports the hypothesis that the switch controls the ATPase activity [42], but experimental verification of this mechanism is still incomplete and our analysis is based on comparisons of structures that are quite divergent in sequence so we cannot be sure of their functional equivalence. Further biochemical and structural data, particularly for the same clamp loader captured in different states of the cycle, are necessary to test this hypothesis.

In addition to the state fully bound by ATP, a ternary complex of T4 clamp loader, DNA, and clamp has been crystallized in a state in which the clamp loader B subunit is bound to ADP while the other active sites are bound to an ATP analog [60]. Thus, this structure, which was obtained adventitiously, represents a state in which only one ATPase site has hydrolyzed ATP. The release of the A and B ATPase from the rest of the ATPase subunits, as a consequence of this ATP hydrolysis, shows that a conformational change within the AAA+ module caused by ATP hydrolysis is incompatible with the symmetric AAA+ spiral.

The AAA+ spiral probably comes apart in a cooperative fashion, starting from one end of the spiral (Figure 6). ATP hydrolysis is cooperative in the presence of primer template DNA [98]. Therefore, hydrolysis at one site in the clamp loader will promote hydrolysis at another site. We also suspect that the cooperative disassembly of the AAA+ spiral will be directional, starting from the ends of the spiral, because ATP hydrolysis requires movement of neighboring subunits away from each other. This movement will be favored at an end of the spiral, as this order imposes changes to only one subunit for each hydrolysis event. Biochemical studies [45,99] and structural data [60] corroborate this hypothesis. Hydrolysis from the ends has the functional benefit of allowing the clamp to close before the clamp loader is fully ejected, which would prevent the DNA from slipping out from the opening of the clamp before the loading reaction is complete. In support of this idea, the clamp loader with ADP at the B site is bound to a closed clamp in the structure, which is a result of the breakdown in the symmetric matching of the clamp loader's binding sites with those of both the DNA and the sliding clamp [60]. Thus, upon further ATP hydrolysis events at the C and/or D sites, the clamp loader can no longer recognize the symmetrically arrayed binding sites on the clamp and DNA, and therefore ejects from both macromolecular substrates, leaving the primer-template junction threaded through the ring of the clamp (Figure 6).

One apparently consistent feature of clamp loaders from different domains of life is that only three of the ATPase subunits are catalytically active in clamp loading. (Mutated clamp loader complexes with fewer than three active ATPase sites can accomplish clamp loading [46,100,101], but only with greatly diminished efficiency.) In the case of bacterial clamp loaders, it is clear that only the B, C, and D subunits are active ATPases. The eukaryotic clamp loaders contain five subunits that can bind ATP, but activity appears to be necessary only in the B, C, and D subunits. The E site can bind nucleotide [59], but lacks catalytic activity. The A subunit can both bind and hydrolyze ATP, but its activity is not necessary for clamp loading in either the eukaryotic [102] or archaeal clamp loader [63]. Likewise, in T4, the identical B, C, D and E subunits all have ATP binding sites, but the E subunit appears to be catalytically inactive, because the A' domain does not contribute an arginine finger residue (normally supplied in *trans*) to complete the catalytic machinery [60]. However, there is some controversy regarding the stoichiometry of ATP usage in the T4 clamp loading cycle [94,98,103-105].

## The T4 bacteriophage replication system seems to be a chimera, with functional modules borrowed from bacteria and eukaryotes

**Figure 7:**
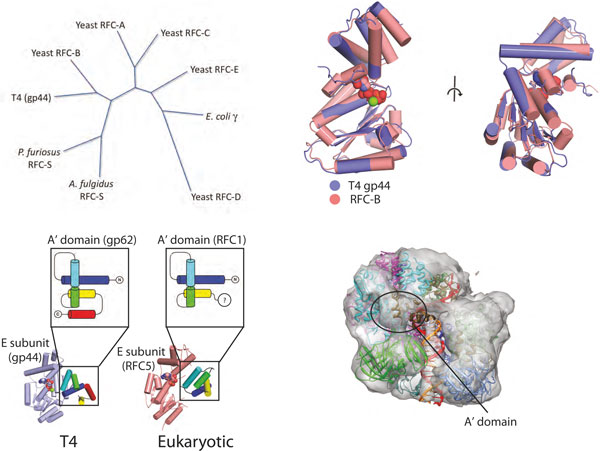
**Sequence and structural relationships between the T4 clamp loader and those from other branches of life**. **(a) **A sequence similarity dendrogram for the structurally characterized clamp loader subunits. The T4 clamp loader is more similar to yeast clamp loader than to the bacterial clamp loader subunits. Tree calculated using PHYLIP [133] using the neighbor-joining bootstrap criterion. **(b) **The structure of the T4 clamp loader B position AAA+ module (the gp44 protein; purple) is highly similar to the yeast clamp loader B subunit AAA+ module (the RFC4 protein; salmon). The C_α _root mean square deviation (RMSD) is approximately 1.1 and 1.4 Å for the amino- and carboxy-terminal domains of the AAA+ modules, respectively. ATP is shown in spacefilling representation. **(c) **The A' domain is similar in yeast and T4 clamp loaders. The T4 and yeast A' domains have identical fold topology (the gp62 and RFC1 proteins in purple and salmon, respectively). Additionally, their packing against the E subunit AAA+ module is very similar. **(d) **A negative stain electron microscopy reconstruction of the *P. furiosus *clamp loader, clamp and DNA complex [62] reveals the presence of an A' domain. The correspondence of the T4 clamp loader structure (fit to the electron microscopy-derived molecular envelope) with that of the archaeal clamp loader suggests that the *P. furiosus *clamp loader also has an A' domain. Figure adapted from [60].

The genome of T4 bacteriophage is a curiosity, in that it is thought to be composed of genes derived from both eukaryotic and bacterial sources [106], even though it infects only bacteria. The hybrid nature of T4 is further supported by the evidence that double-stranded DNA bacteriophages share similarity in their capsid proteins [107-109] as well as DNA packaging machinery [110,111] with eukaryotic viruses such as herpesviruses. In fact, many eukaryotic viruses seem to have a hybrid genome, with significant horizontal transfer of bacterial and archaeal genes [112,113]. In the case of T4, the similarity of T4 proteins to their eukaryotic counterparts is apparent from the sequence and structures of the clamp and clamp loader. Both eukaryotic and T4 clamps are homotrimers, unlike the dimeric bacterial clamps, and the T4 clamp loader AAA+ subunit shows more sequence homology to eukaryotic clamp loaders than to bacterial clamp loading subunits (Figure 7a): this sequence homology is reflected in the high structural similarity of the B, C, D and E subunits of T4 with those of the yeast clamp loader (Figure 7b).

One of the unique features of both the eukaryotic and T4 clamp loaders is the A subunit. The T4 A subunit forms an inverted U shape and reaches across the clamp interface [60]. Therefore, the A subunit binds two clamp subunits, with the A domain binding to the clamp I subunit and the A' domain binding to the clamp III subunit (Figures 4c and 5b). We propose that the A subunit of the eukaryotic clamp loader is also likely to bind to both the clamp I and the clamp III subunits. The yeast clamp loader structure shows that the A subunit also forms an inverted U, although the A' domain has collapsed down onto the AAA+ module of the A subunit, and there is no contact between the A' domain and the clamp [59]. This collapse probably resulted from mutations in the clamp loader that prevented clamp opening, or deletion of the carboxy-terminal portion of the A' domain, or both. Whatever the evolutionary history, the yeast and T4 A' domains interact with the E subunit in similar ways and have essentially the same fold (Figure 7c). Furthermore, electron microscopic reconstruction of the *Pyrococcus furiosus *clamp loader demonstrates that archaeal clamp loaders also have the A' domain and shows electron density that reaches across the interface and touches the clamp, like the T4 loader [60,62] (Figure 7d) but unlike the *E. coli *A subunit, which binds only one protomer of the clamp and contains no A' domain [32,114].

These findings may bear on the question of how the oligomeric character of the clamp influences the structure and mechanism of its loader. In particular, the trimeric clamps of T4 and eukaryotes are less stable [85], and thus may require a subunit to bind both sides of the open interface to help stabilize the open form and to keep clamp protomers from dissociating. In contrast, bacterial clamps are dimeric and appear to be the most stable [85], perhaps eliminating a requirement for additional stabilization of the open form by the A' domain.

The similarity between the eukaryotic and T4 bacteriophage clamp loaders may reflect a deeper shared evolutionary history. The T4 DNA polymerase belongs to the B family, as do the eukaryotic replicases [115], whereas the bacterial replicases are members of the unrelated C-family of polymerases [116,117]. In other components of the replisome, however, there are significant similarities between T4 components and those of bacteria. The T4 and *E. coli *primases are single-subunit enzymes [118-121] and both have TOPRIM folds [122-124]. In contrast, the eukaryotic primase (Pol α) is a four subunit assembly containing both a primase activity and a DNA polymerase that extends the RNA to make a chimeric RNA-DNA primer [125,126]. The T4 helicase is related to the bacterial replicative helicase (*E. coli *DnaB) [122] and has the same directionality of unwinding as *E. coli *DnaB, implying that these helicases encircle the lagging strand at a replication fork [125]. By contrast, the archaeal and eukaryotic MCM hexamers translocate in the opposite direction [127-130], implying they surround the leading strand. Additionally, the single-stranded DNA binding proteins from T4 (gp32) and *E. coli *(SSB) have been suggested to be related [106], although there are significant functional differences in their mechanisms of action. Finally, the T4 gp69 protein, which has been suggested to assist in the initiation of DNA replication [106], has significant homology to the *E. coli *DnaA protein that initiates DNA replication [131].

These results imply that replisomes can be pieced together from different modules. The helicase, primase and single-stranded binding protein of T4 are most closely related to those of bacteria, while the clamp, clamp loader and polymerase are similar to those of eukaryotes. These two groups of proteins represent different functional modules of the replisome: the helicase and primase travel on the lagging strand as one unit, while the polymerase and clamp are tightly associated. These observations suggest that entire modules may be evolutionarily exchanged more readily than individual proteins.

## Open questions

We have now reached a satisfactory state of understanding regarding the structural basis for clamp loader action, at least at the level of the general mechanism, understood in broad strokes (Figure 6). We know what these assemblies look like when they are alone, with and without ATP bound [114,132], and how their structure converts to a tightly integrated spiral form upon binding ATP [55,59,60,62]. The structures have explained how the primer-template junction is recognized [59], and how the clamp loader can accommodate both RNA and DNA primers [55,60]. The recently determined structures of the T4 clamp loader have shown how the ATP-bound clamp loader stabilizes an open form of the clamp, and how ATP hydrolysis might be coupled to release of the clamp onto DNA [60].

Although the inferences drawn from these structures are compelling, it is important to recognize that we only have one structure for some of the key steps in the clamp loading cycle, and that these structures are for clamp loaders that are very divergent in sequence. The structure of the ATP-free clamp loader is for the *E. coli *system [114] (Figure 4a), as is the ATP-loaded form bound to DNA [55]. A structure of the loader in complex with a closed clamp in the absence of DNA is for the eukaryotic clamp loader [59] (Figure 4b), and the complex with the open clamp and DNA is for the T4 complex [60] (Figure 4c). A more comprehensive understanding of how ATP binding and hydrolysis is coupled to the opening of the clamp and its loading on DNA requires that we have structures of the same clamp loader (or, at least, very similar clamp loaders) in different states of the clamp loading cycle. It is hoped that the considerable information now available about the general nature of the conformational changes that are intrinsic to clamp loader function will allow such structures to be obtained in the near future. In addition, it is hoped that a clamp loader bound to an open clamp in the absence of DNA can also be crystallized, which will provide information about how much the clamp is opened before it is loaded on to DNA.

Although we have emphasized the clamp loading aspect of the clamp loader machine, clamp loaders are critical for the proper coordination of leading and lagging strand synthesis. This is most clearly evident in bacteria, where the clamp loaders are physically attached to the polymerase subunits that replicate the leading strand continuously and those that cycle between Okazaki fragments on the lagging strand. The greater challenge ahead is to understand how clamp loaders serve to orchestrate rapid replication of chromosomal DNA, and the bacterial replisome is likely to provide the route to clearer structural understanding, because of the depth of biochemical information available, as well as the ability to purify intact complexes in reasonable amounts. We look forward to future advances in the structural analysis of intact replisome assemblies.

## Supplementary Material

Additional file 1 **The structure of the clamp loader of *E. coli *(known as the γ-complex) in the apo form (PDB code 1JR3)**. Click here for file

Additional file 2 **The structure of the budding yeast clamp loader, replication factor-C (RFC), bound to the sliding clamp, PCNA, and an ATP analog (PDB code 1SXJ)**. Click here for file

Additional file 3 **Movie of the T4 bacteriophage clamp loader bound to primer-template DNA, an ATP analog, and the sliding clamp during clamp closure (PDB code 3U60)**. Click here for file
